# Supplementary figures and images for: Comparative analysis of heparin affecting the biochemical properties of chicken and murine prion proteins
Source: PLoS One. 2021 Feb 18;16(2):e0247248. doi: 10.1371/journal.pone.0247248 (PMC7891698; doi:10.1371/journal.pone.0247248)

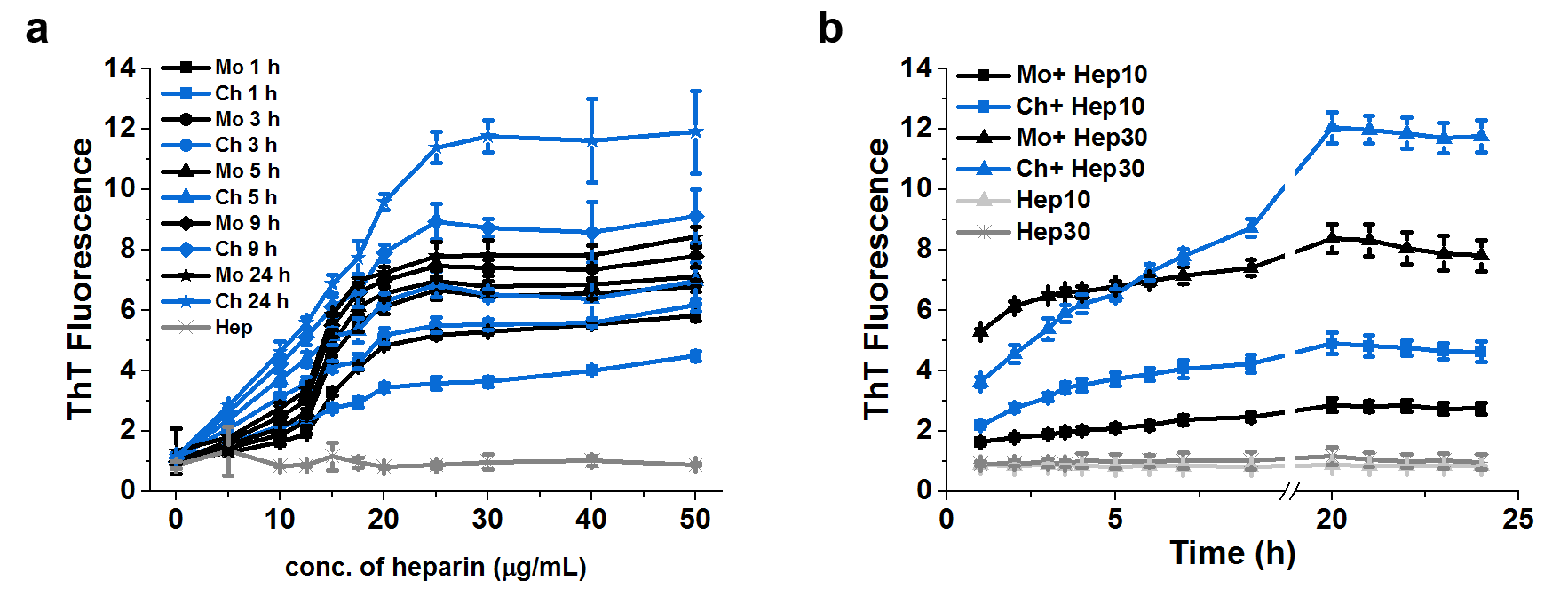

Supplement: S1 Fig — a, ThT fluorescences of PrP, 5 μM ChPrP (blue) or MoPrP (black), in the presence of increasing concentrations of heparin were detected at different times. ThT fluorescences of increasing concentrations of heparin in MilliQ water (light gray) were measured as control. b, the time-dependent changes in ThT fluorescence were determined for PrP (5 μM) after the addition of heparin at 10 μg/mL (square) or 30 μg/mL (triangle). Those of 10 μg/mL or 30 μg/mL heparin in solutions of MilliQ water alone (light gray and dark gray respectively) were also measured as controls. Error bars are the standard deviation (SD) of at least 3 repeats and are smaller than the symbol when absent in the figure. (TIF) [file pone.0247248.s001.tif]

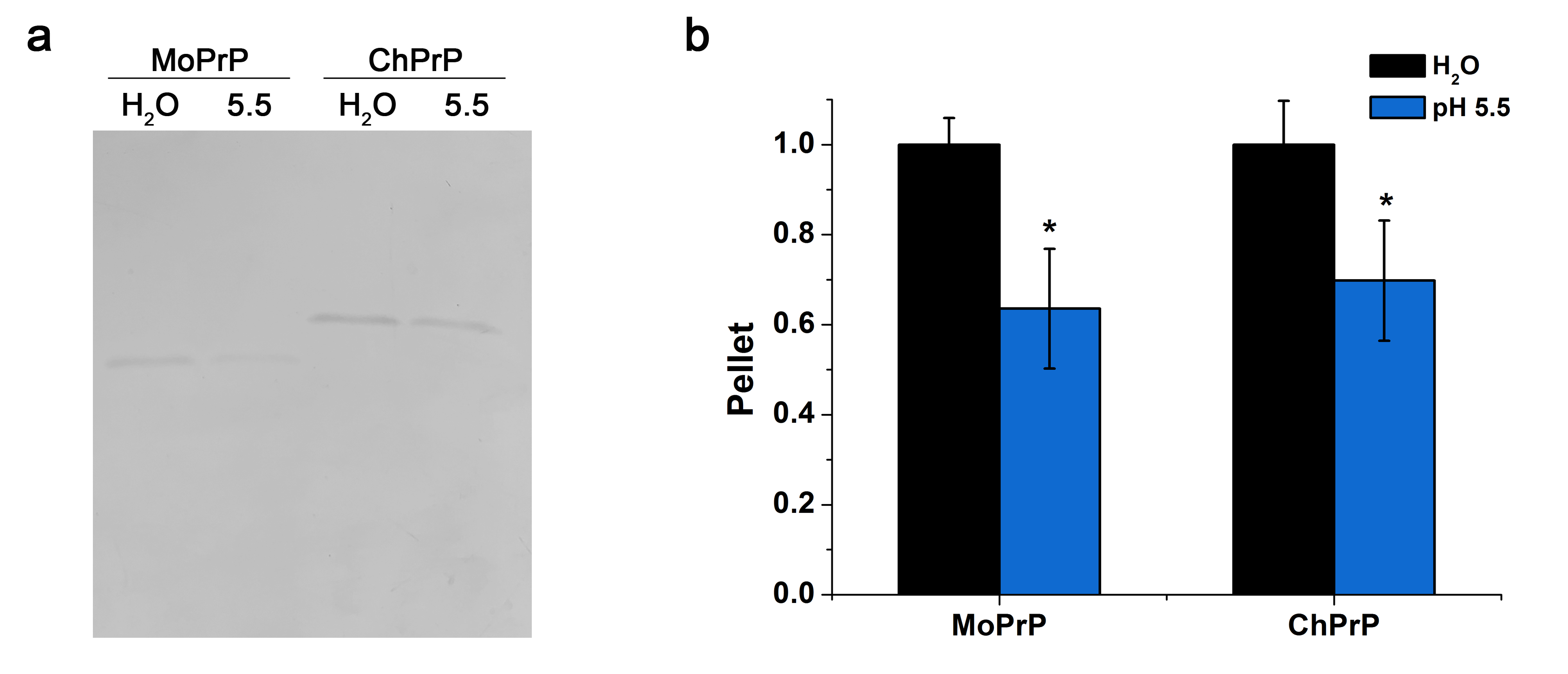

Supplement: S2 Fig — The PrP at 5 μM was incubated in MilliQ water or in buffer pH 5.5 for 24 h at 25°C and then centrifugated for 10 min at 13,000 × g. Supernatant was separated from the pellet; the pellet was then resuspended in water to the same volume as the supernatant. The pellet samples were subjected to SDS-PAGE, visualized using Coomassie staining (a) and quantified using ImageJ software (b). The columns in panel b show the fold changes relative to the pellet portion of PrP in water. *P < 0.05 (Student’s two tailed t-test), n = 3, mean ± SD. (TIF) [file pone.0247248.s002.tif]

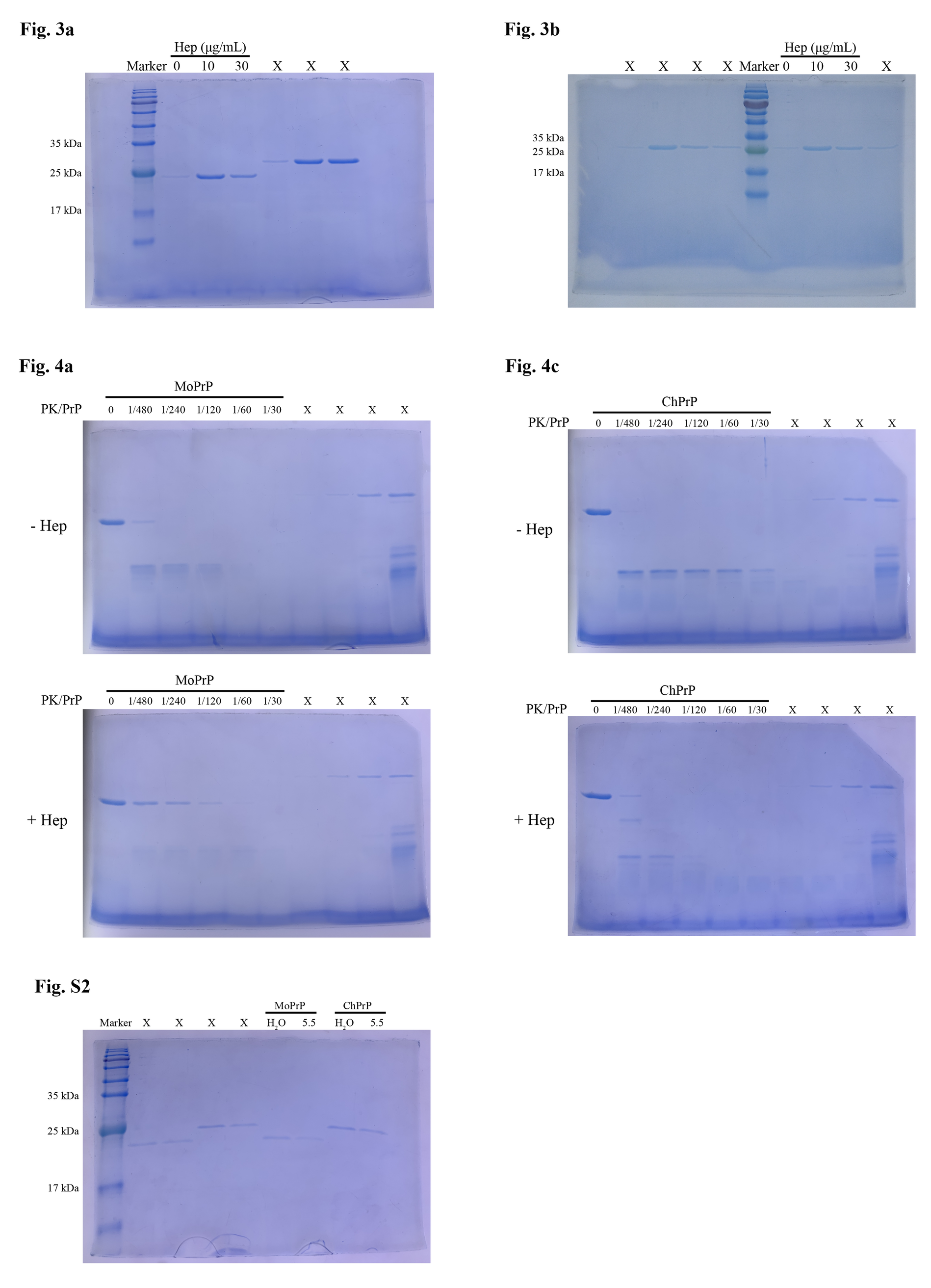

Supplement: S1 Raw images — (TIF) [file pone.0247248.s003.tif]
