# Supplementary material for: Comparative analysis of heparin affecting the biochemical properties of chicken and murine prion proteins
Source: PLoS One. 2021 Feb 18;16(2):e0247248. doi: 10.1371/journal.pone.0247248 (PMC7891698; doi:10.1371/journal.pone.0247248)
Supplement: Attachment — Submitted filename: Reviewer Recommendation and Comments for Manuscript Number PONE-D-20-18047.docx [file pone.0247248.s004.docx]

1. Summary of the research and your overall impression

The authors biochemically and biophysically investigated the changes between a species of prion protein known to misfold into a disease associated form and a species not shown to be capable of this structural change when they are incubated with a potential cofactor mimetic.

Mouse PrP is known to be capable of undergoing conformational change to PrPSc and even recombinant MoPrP can instigate an infectious prion disease when combined with the right cofactors. This makes this protein a very sensible choice by the authors for this investigation. They also chose to use Chicken PrP as a non-disease-causing prion protein variant as prion diseases are mammalian diseases and are not known to affect birds. This enabled comparison between two protein homologues to investigate cofactor driven misfolding properties of PrP which make mammals victims of this disease.

Well established indicators of disease-associated misfolding were examined including fibril formation, ThT reactivity, solubility, Proteinase K resistance, increased beta-sheet secondary structure and aggregation. The addition of Heparin to MoPrP shifted the proteins properties to become more insoluble, aggregation prone, PK resistance and to possibly have a greater beta-sheet content when compared to untreated MoPrP, and ChPrP with and without Heparin treatment. MoPrP more readily forms ThT positive species under fibril generating conditions than ChPrP with Heparin not shown to contribute to this phenomenon.

The objective of this paper was to see if the prion protein from a class of animals susceptible to prion disease is more likely to develop disease associated biochemical and biophysical features both alone and in the presence of a potential cofactor than PrP from a class of animals resistant to prion disease and this objective was met but there are significant issues which need to be addressed. This paper also highlights an important property of prion protein behaviour in that it is very susceptible to concentration/molar ratio of cofactors. PrP behaviour can vary significantly with changes in treatment concentration. This review supports the publication of this data if the follow major issues are addressed adequately.

2. Discussion of specific areas for improvement

Line 51-58

The authors explain their use of Heparin as a mimetic for Heparan Sulfate by discussing its ability to generate prions in PMCA (ref 18) and its history of use as a mimetic. It is disappointing that the authors didn’t mention the findings of ref 19 that the lower levels of sulfation if Heparan Sulfate change the way PrP behaves biochemically when compared to Heparin. The authors will need to give an explanation of their decision to use Heparin in a way which is relevant to their specific study given that many experiments mirror those of ref 19.

Line 179-182

It is disappointing that the data showing the proportion of soluble to non-soluble PrP was not included. Can the authors state a reason for why they chose to include comparisons of pellet intensity (Fig 3) and not soluble vs insoluble PrP with and without Heparin treatment? Also, my understanding of agreeing to the statement “Yes - all data are fully available without restriction” implies you can’t have “data not shown”. This needs to be rectified

Line 215-218

The authors claim that the EM data for ChPrP treated with Heparin supports the small decrease in amplitude in the CD spectra. I don’t believe it is possible to draw such a conclusion without further experimental evidence. The size of aggregates under EM is unrelated to the secondary structure detected by CD.

Line 252-254

It is interesting to state that PrP is more soluble in sodium acetate buffer than water but inappropriate to bring new data into the Discussion section. S2 needs to be moved to the results section. The same comment applies as above - Can the authors state a reason for why they chose to include comparisons of pellet intensity and not soluble vs insoluble PrP? Can they also explain why they chose not to examine solubility during Heparin treatment in sodium acetate buffer?

Figure 3a + b

The blots are very poor quality and seem to be doctored. Can the authors provide raw, high quality files for these blots?

Figure 3c + d

y-axis should both have the same scale to allow easy comparison of MoPrP and ChPrP. Also, the y-axis label is insufficient and should mention that it is a fold increase over untreated pellet

Figure 4a + b

The blots are very poor quality. Can the authors provide raw, high quality files for these blots?

Figure 4 c + d

Can the authors explain why they chose two different populations of protein bands to quantify for these western blots? For this research question it would seem more appropriate to either quantify full length protein for both MoPrP and ChPrP or to quantify all bands present.

Figure 5a

MoPrP solubility with Heparin seems particularly poor in these researchers hands as the ellipticity is low and ragged at both Heparin concentrations. It is difficult to confidently claim Heparin induced beta-sheet structure in MoPrP from these results.

Figure 6 c and g

Images are very poor quality and seem out of focus. Can the authors provide higher quality, in focus images? It is also unusual that given how insoluble the PrP became upon addition of 10ug/ml of heparin that there were no aggregates seen in the EM images – can the authors provide a comment on these observations?

Grammatical changes:

Overall, the use of PrPs to describe the samples used should be changed to either PrP or recPrP – PrP does not need to be pluralised with an s.

Line 34

Change to “…protein, PrP^Sc^, and without”

Line 35

Remove the word “obviously”

Line 36

Remove the word “the”

Line 37

Change to “aggregates”

Line 38

Change to “studies” not ‘researchers’

Line 41

Remove the word “However”

Line 43

Change from “only PrPSc” to “PrPSc alone”

Line 47

‘Characters’ is not the right word – try ‘molecules’ instead

Line 60

Change to “Causes prion diseases”

Line 71

Change to “…about the effects”

Line 129

Remove “the signal value of”

Line 130-131

Change to …or not, both recPrP species displayed…”

Line 133

Change to “under denaturing conditions”

Line 139-140

Change to “When either species of rec PrP in MilliQ…”

Line 158

Change to “form ThT positive aggregates”

Line 167

Change to …” a low intensity, full length MoPrP band was detected…”

Line 182

Remove “of PrPs”

Line 211

Change ‘much’ to ‘many’

Line 223-224

Change to “the conversion of mammalian PrP into protease-resistant forms”

Line 228

Change ‘that on’ to ‘those of’

Line 235

Change to ‘denaturing conditions’ and change ‘binding’ to ‘addition’

Line 266

Change to “directly influences the properties of PrP”

Line 500

Change to “Role of heparin treatment on the solubility of recPrP”

3. Any other points

I am happy to look over any revised manuscript
