# Supplementary material for: Comparative analysis of heparin affecting the biochemical properties of chicken and murine prion proteins
Source: PLoS One. 2021 Feb 18;16(2):e0247248. doi: 10.1371/journal.pone.0247248 (PMC7891698; doi:10.1371/journal.pone.0247248)
Supplement: Attachment — Submitted filename: Response to Reviewers.docx [file pone.0247248.s006.docx]

**Listed Responses to the comments from the editors and reviewers**

Dear Byron Caughey and Dear Reviewers:

Thank you for your efforts to handle and review our revised manuscript (PONE-D-20-18047_R1) entitled: “Comparative analysis of heparin affecting the biochemical properties of chicken and murine prion proteins”. We have carefully revised the manuscript based on your suggestions. Followings are Listed Responses to the Editor and Reviewers’ Comments and Suggestions:

Thank you for submitting your manuscript to PLOS ONE. After careful consideration, we feel that it has merit but does not fully meet PLOS ONE’s publication criteria as it currently stands. One of the reviewers still has major concerns about your manuscript that I would ask you to address if possible in a second revision.

Response: We have addressed the reviewers' comments carefully below. We appreciate the Editor for the opportunity.

Reviewer #1: I am satisfied with the improvements made by the authors to this manuscript. I acknowledge their hard work and look forward to their future studies.

Response: Thank you for your positive comments.

Reviewer #2: Dear Authors,

I still have important criticisms about the data and interpretations presented in the paper “Comparative analysis of heparin affecting the biochemical properties of chicken and murine prion proteins”, and for this reason, I forward my observations for your appreciation. Here are the first observations made by me, with the authors' response and my counterarguments.

Response: Thank you for your comments. We appreciate the reviewer’s efforts to read this paper and previous paper. We have addressed the review’s concerns point by point below.

1 - The experiments in figure 2 used ThT to follow PrP aggregation. However, the fiber morphology by TEM, and the CD signal are not characteristic of fibrillar forms. PrP:Hep 30 ug/mL samples showed the highest ThT fluorescence, but the smallest pellet. The authors should address and explain this contradiction. I suggest checking if the light scattering signal is not interfering with ThT signal. The authors should also show the ThT spectra of the samples with great variation to discard this doubt. The author could also use other approaches to confirm fibril formation.

Author Response: Thank you for your good suggestions. In figure 1, we studied the effects of heparin on fibril formation of PrP and found that heparin may not play a crucial part in amyloid fibril formation under denaturing conditions. In the rest of the manuscript, we did not study the formation and characteristics of fibers. The CD and TEM were used to monitor the conformational changes and aggregates of PrP. Indeed, PrP:Hep 30 μg/mL samples showed the highest ThT fluorescence, but the smallest pellet. However, the protein in the pellet did not have to be aggregated and electron micrographs of resuspended pellet showed no significant aggregates. Protein aggregation into amyloid fibrils is a pathological hall-mark of many diseases, including prion. We would investigate fibril formation in detail in our future studies. Thanks very much for the reviewer’s good suggestion again.

Reviewer Response: although the authors answered that they evaluated the formation of fibers only at Fig1, they used ThT in Fig2 and observed the morphology of aggregates in Fig6. There are serious inconsistencies between the results observed in Fig 2, 3, 5 and 6. The authors argue that although showing the highest ThT signal, the sample in the presence of 30 ug/mL of Heparin forms a smaller pellet (Fig3), because “protein in the pellet did not have to be aggregated and electron micrographs of resuspended pellet showed no significant aggregates".

The first problem of this answer is that the protein being studied will only form a pellet after centrifugation for 10 min at 14000 g (line 119) if it is in an oligomeric/aggregated state.

Response: Thanks for your suggestions. It is really true as the reviewer suggested that the protein being studied will only form a pellet after centrifugation if it is in an oligomeric/aggregated state. Sorry for the confuse. What I mean is the protein in the pellet did not have to undergo a conformational change. PrP:Hep 30 μg/mL samples showed the highest ThT fluorescence, but the smallest pellet. In contrast, PrP:Hep 10 μg/mL samples showed lower ThT fluorescence, but the more pellet. Such results have previously been reported (Ellett LJ, et al, 2015, Glycobiology. Doi: 10.1093/glycob/cwv014.). HS treated recMoPrP showed visible turbidity, whereas the ThT fluorescence of those were low. The contradiction might be explained by that the protein in the pellet did not have to undergo a conformational change.

The second problem of this answer is that Fig 6 shows significant aggregates for MoPrP+Hep30. The aggregate state is not directly related to the fiber morphology, not having a direct relationship with the observed signal. However, since there is ThT fluorescence and pellet, the authors should observe the morphology of protofibers and/or fibers in this electron micrographs. Author should explain this contradiction or show that the sample has soluble fibers that did not go to the pellet.

Response: Thanks for your good suggestions. Indeed, MoPrP+Hep30 showed high highest ThT fluorescence. The increase of ThT fluorescence suggests a conformational change, but does not necessarily imply fiber formation. In addition, the experiments of Fig 2 to Fig 6 were all performed under physiological conditions, whereas amyloid fibrils were usually formed under denaturing conditions. Thus, we didn’t observe the morphology of protofibers and/or fibers in electron micrographs. Similar results were observed in Ellett LJ, et al, 2015, Glycobiology. Doi: 10.1093/glycob/cwv014. Only aggregates were observed when recMoPrP was incubated with GAGs under physiological conditions.

The authors reported that the PrP+Hep10 sample was almost entirely going to the pellet (although they have not yet shown the relationship of how much remained in the supernatant at this and the other conditions studied) (Fig3). However, this PrP:Hep sample showed the greatest sign of ellipticity (Fig 5) and was less aggregated in the electron micrograph (Fig 6), suggesting its less aggregated. The authors should discuss this.

Response: Thanks for your good suggestions. Pellet fraction of MoPrP+Hep10 and ChPrP+Hep10 samples were approximately 75%~ 80% (Fig 3). The pellet was separated by centrifugation (14,000 g, 30 min) and the supernatant was used for CD analysis. The CD spectra was significantly lost (almost changed to smooth curves) when 10 μg/mL heparin was used (Fig 5), which was consistent with the formation of insoluble aggregates (visible turbidity). Electron micrographs of PrP+Hep10 samples showed no significant aggregates. Electron micrographs of resuspended pellet showed no significant aggregates, either. The protein pellet could not attach well to the copper grids, whereas the oligomeric protein could. This might explain why PrP+Hep10 samples were less aggregated.

2 - PK treatment was performed in order to show if the aggregates formed were resistant, “Resistance to digestion with PK is one of the characteristics of PrP fibrils and PrPSc” (line 178 and 179).
Figure 4 shows this data, but figures are not of good quality as protein is degraded by PK at concentrations lower than usually used for PrP fibrils or PrPSc. PrP:Hep 30 ug/mL was used, although it was less aggregated than PrP:Hep 10 ug/mL (Figure 3). ChPrP was more resistant to PK alone than MoPrP and both samples with Hep, although ChPrP:Hep 30 ug/mL showed the highest ThT fluorescence (Figure 1). Authors must clarify and discuss these contradictions. Also, authors should include the acrylamide concentration in the MM section.

Author Response: Thank you for your good suggestions. In Fig 4, PrP was incubated with heparin in MilliQ water without any seeds, which was not a suitable condition for fibril formation. Under this condition, the interaction between PrP and cofactor causes widespread and persistent conformational changes to form an intermediate species of PrPSc (A. Zurawel, et al. Biochemistry, 2014, doi: 10.1021/bi4014825). These may be the reason why PK concentrations used in Fig 4 were lower than usually used for PrP fibrils or PrPSc. Indeed, PrP incubated with low concentration of heparin (10 μg/mL) were less soluble than those treated with heparin at high concentration (30 μg/mL) (Fig 3). However, the protein in the pellet did not have to be aggregated. The presence of pellet did not imply conformational changes, and most of the protein in the pellet is not conducive to subsequent experiments. Thus, 30 μg/mL heparin was used when we analyzed the PK resistance. ChPrP:Hep 30 μg/mL showed the highest ThT fluorescence (Fig 1), which suggests conformational changes were occurred after incubation with heparin. However, PK-resistant form may not be the only destination of the conformational conversion. It may be the reason why ChPrP was more resistant to PK alone than ChPrP with Hep. Sorry for not including the acrylamide concentration in the MM section. The acrylamide concentration (12%) was used in this study. Details were added to the revised manuscript at Page 6 Lines 109-111 and Page 7 Lines 115-116.

Reviewer Response: When the authors answer “These may be the reason why PK concentrations used in Fig 4 were lower than usually used for PrP fibrils or PrPSc” they should discuss it in the paper for readers. The authors answered “However, the protein in the pellet did not have to be aggregated. The presence of pellet did not imply conformational changes, and most of the protein in the pellet is not conducive to subsequent experiments”. Again, over the centrifugation performed, the presence of the monomeric form of the protein forming the pellet would be very unlikely, which would have to be shown, although the authors are right when they say that there is not necessarily a conformational change, which would have to be shown as well. But once there is a ThT fluorescence increment (Fig 1), there is a suggestion of conformational change.

I agree when the authors wrote “However, PK-resistant form may not be the only destination of the conformational conversion” and they should discuss this for readers. But, when they wrote “It may be the reason why ChPrP was more resistant to PK alone than ChPrP with Hep”, this argument is very unconvincing.

Response: Thanks for your good suggestions. We have added additional discussion on why PK concentrations used in Fig 4 were lower than usually used for PrP fibrils or PrP^Sc^. More details had been added to the revised manuscript at Page 17 Lines 326-332. The revised parts were as followings: Heparin-treated MoPrP was more PK resistant, whereas the MoPrP was degraded by PK at concentrations lower than usually used for PrP fibrils or PrP^Sc^. Here, PrP was incubated with heparin in MilliQ water without any seeds, which was not a suitable condition for fibril formation. Under this condition, the interaction between PrP and heparin causes widespread and persistent conformational changes to form an intermediate species of PrP^Sc^ [46]. These may be the reason why PK concentrations used here were lower than usually used for PrP fibrils or PrP^Sc^.

Sorry for the confuse. After incubating of PrP and heparin, centrifugation was performed only before CD, whereas other experiments, including TEM, were performed without centrifugation. To avoid confusion, we revised the description of TEM method. More details had been added to the revised manuscript at Page 7 Lines 122-127. The revised parts were as followings: The pellet was separated by centrifugation (14,000 *g*, 30 min) and the supernatant was used for CD analysis. The ellipticity values (MilliQ water or heparin solution) were used as controls. For TEM, 4 μL incubating solution not centrifuged was fixed on 300 mesh copper grids (BZ10023b, Zhongjingkeyi), washed with 4 μL water, negatively stained using 2% uranyl acetate and examined on a Tecnai G2 Spirit TEM at voltage of 120 kV.

It is really true as the reviewer suggested that “once there is a ThT fluorescence increment (Fig 1), there is a suggestion of conformational change.” Protein conformational changes might be masked by aggregates in CD. FTIR would be more appropriate in this case, whereas this instrument is not available in our university at present. If there is an opportunity in the future, we will definitely consider using FTIR for investigating secondary structures.

We have added additional discussion and deleted the unconvincing sentences as the reviewer suggested. More details had been added to the revised manuscript at Page 17 Lines 332-338. The revised parts were as followings: In contrast, ChPrP was more resistant to PK alone than ChPrP with 30 μg/mL heparin. The ChPrP treated with 30 μg/mL heparin showed the highest ThT fluorescence (Fig 2b), which suggests conformational changes were occurred after incubation with heparin. The formation of β-sheet conformation in prion protein usually leads to a protease-resistant form [19,20]. However, PK-resistant form may not be the only destination of the conformational conversion [47].

3 - The authors used CD spectroscopy to show changes of PrP secondary structure. “When MoPrP was treated with 30 μg/mL heparin, a dramatic decrease in the amplitude of the CD spectrum is observed (Fig 5a), the associated β-sheet content of which was 13.8-fold increase compared to MoPrP in solution. In contrast, incubating of the ChPrP to 30 μg/mL heparin induces a relatively small decrease in the amplitude of the CD spectrum (Fig 5b), and only 1.3-fold increase in the β-sheet content was estimated relative to the secondary structure of ChPrP in solution” (lines 199-205).
The authors did not describe how they calculated the beta sheet content, and whether they used any spectrum deconvolution. A visual analysis of the result is not sufficient for this result. The data obtained with ChPrP + Hep 10 was not described. In addition, the MoPrP + Hep 30 data does not show enough ellipticity signal to carry out deconvolution. As the authors point out, the CD is an absorptive technique and changes in sample turbidity impair the analysis of secondary structure content. Another technique like FTIR would be more appropriate in this case. The dichroism data seems to corroborate with a greater aggregation and, therefore, a greater loss of signal, as observed in the TEM data of figure 6, but this does not allow inference about changes in the protein secondary structure. However, the data shows a different effect between murine and chicken proteins.

Author Response: Thank you for your good suggestions. The β-sheet content was calculated by the software of the chiascan spectropolarimeter (Applied photophysics). It is really true as the reviewer suggested that the MoPrP + Hep 30 data does not show enough ellipticity signal, thus we deleted the calculated β-sheet content of MoPrP+ Hep 30. Details could be found in the revised manuscript at Page 25 Lines 491-494. The data obtained with ChPrP + Hep 10 was described in the revised manuscript at Page 13 Lines 245-247. It is really true as the reviewer suggested that FTIR would be more appropriate in this case, whereas this instrument is not available in our university at present. If there is an opportunity in the future, we will definitely consider using FTIR for investigating secondary structures. In this study, it was intended to compare the different effects of heparin on ChPrP and MoPrP. Although changes in sample turbidity impair the analysis of secondary structure content using CD, different effect of 30 μg/mL heparin on murine and chicken proteins was observed.

Reviewer Response: The last version of the manuscript still has sentences about calculated beta sheet content as “In contrast, incubating of the ChPrP to 30 μg/mL heparin induces a relatively small decrease in the amplitude of the CD spectrum (Fig 5b), and only 1.3-fold increase in the β-sheet content was estimated relative to the secondary structure of ChPrP in solution.” The previous sentence says “When MoPrP was treated with 30 μg/mL heparin, a dramatic decrease in the amplitude of the CD spectrum is observed (Fig 5a)”, so they are describing different effects for the same sample.

Response: Thanks for your good suggestions. We have deleted the sentences about calculated beta sheet content. And we described the effects of 30 μg/mL heparin on different samples (MoPrP and ChPrP) in our last version of the manuscript. Fig 5a and Fig 5b show the CD spectra of MoPrP (a) and ChPrP (b), respectively.

4 - “As for ChPrP treated with 30 μg/mL heparin, less PK resistance and slight increase of β-sheet structure was observed, suggesting that no significant conformational changes occurred and the infectious form PrPSc may not be the only destination of the conformational conversion [47, 48].” (lines 267-270). Conclusions like this must be reconsidered and rephrased.

Responses: Thanks for your suggestion. We revised the related sentences to delete the inappropriate speculation. The revised parts can be found in the revised manuscript at Page 17 Lines 329-330.

Reviewer Response: ok

Response: Thank you for your positive comments.

Minor comments:

1- Running title: I suggest changing for a more specific running title.

Author Response: Thanks for your suggestion. We have changed the running title to “Effect of heparin on ChPrP and MoPrP aggregation”.

Reviewer Response: ok

Response: Thank you for your positive comments.

2 - All data were obtained in water or sodium acetate buffer, with no NaCl. The interaction of Hep with PrP is electrostatic, modulated by salt concentration. No NaCl may enhance the aggregation induced by Hep. Absence of NaCl can also mask possible differences between affinity for murine and chicken PrP. I suggest performing the experiment over different NaCl concentrations, comparing with the literature. Indeed, the effect showed to be different between the two PrP constructs.

Responses: Thanks for your good suggestion. In our current study, Thioflavin T fluorescence of PrP was measured both in MilliQ water and sodium acetate buffer. The results were consistent in both solutions. To avoid the impact of buffers, subsequent experiments are carried out in the MilliQ water. Indeed, the effect was showed to be different between the two PrP constructs. Thanks very much for the reviewer’s good suggestion, which gave us more inspiration to study the differences between affinity of murine and chicken PrP to cofactors, which would be carried out in detail in our future studies.

Reviewer Response: Author should include this perspective and its importance in discussion for the readers.

Response: Thank you for your good suggestions. We have added additional discussion to the revised manuscript at Page 16 Lines 314-318. The added parts were as followings: Additionally, the electrostatic interaction of heparin with PrP might be modulated by salt concentration. In the current study, the reaction solutions with no NaCl might mask possible differences between affinity for MoPrP and ChPrP, which would be carried out in detail in our future studies.

3 - The authors centrifuged PrP:Hep samples and analysed the pellet at Figure 3. In the text its written “almost all PrPs treated with 10 μg/mL heparin were found in the pellet fraction after centrifugation (data not shown)” (line 180 and 181). I suggest showing the data, not only to compare the effect of different concentrations of Hep, but also to show how much of the total protein aggregates.

Responses: Thanks for your good suggestion. We have revised the Fig 3 to support “almost all PrPs treated with 10 μg/mL heparin were found in the pellet fraction after centrifugation” and deleted “data not shown” in the revised manuscript on Page 12 Line 219. The newly revised Fig 3 was as followings:

Reviewer Response: when calculating the fraction present in the pellet of PrP:Hep samples using the densitometry of the band referring to the pellet of PrP alone (which must have negligible amounts of protein in the pellet), the authors do not give us the real dimension of how many of the 5 µM of protein was going to the pellet. This does not invalidate the observation made; however, it would be very enlightening if we had information on how much of the total protein is going into the pellet.

Response: Thanks for your good suggestions. I am so sorry for the mistake in the legend of revised Fig 3 in our last manuscript. The revised Fig 3 showed the pellet fraction of total PrP, not relative to the pellet of PrP in the absence of heparin. And we revised the legend of Fig 3 at Page 11 Line 211-212. The revised parts were as followings: The fraction of PrP in pellet was quantified using ImageJ software and was shown as a percentage of total protein.

Indeed, small amounts of PrP band were detected in pellet fraction of PrP alone after centrifugation, which was shown in Fig 3 (The “0” represents PrP alone, with no heparin).

4 - MoPrP and ChPrP seems to be aggregated before Hep addition. Authors should consider this when analyzing all the data and interpreting the effect of heparin.

Responses: Thanks for your good suggestion. Indeed, a small amount of MoPrP and ChPrP precipitated even in the absence of heparin (Fig 3). Therefore, high speed centrifugation (14,000 g, 4°C, 30 min) was performed before all experiments in this manuscript. Moreover, PrP alone sample, regarded as a control sample, was included in all experiments and the effect of heparin was summarized through comparative analysis of the characteristics of PrP in the absence or presence of heparin.

Reviewer Response: Authors included the sentence “The pellet was separated by centrifugation (14,000 g, 30 min) and the supernatant was used for further analysis” in Circular dichroism (CD) and transmission electron microscopy (TEM) subsection of MM. As it is written, it is giving the impression to the reader that after incubation with the ligand, the protein went through this centrifugation step and the supernatant was analyzed by CD and TEM, but the results should show analysis of the pellet.
Fig 3 and 6 show that PrP alone, even after the centrifugation performed, shows some aggregates. As the authors answered, this sample was correctly used as a control in all experiments. Even so, the authors should consider in the discussion a possible effect of the ligand over the balance between aggregated and non-aggregated species.

Response: Thanks for your good suggestions. Sorry for the confuse. After incubating of PrP and heparin, centrifugation was performed only before CD, whereas other experiments, including TEM, were performed without centrifugation. To avoid confusion, we revised the description of TEM method. More details had been added to the revised manuscript at Page 5 Line 9-21. The revised parts were as followings: The pellet was separated by centrifugation (14,000 *g*, 30 min) and the supernatant was used for CD analysis. The ellipticity values (MilliQ water or heparin solution) were used as controls. For TEM, 4 μL incubating solution not centrifuged was fixed on 300 mesh copper grids (BZ10023b, Zhongjingkeyi), washed with 4 μL water, negatively stained using 2% uranyl acetate and examined on a Tecnai G2 Spirit TEM at voltage of 120 kV.

PrP alone sample, regarded as a control sample, was included in all experiments. The possible effect of heparin over the balance between aggregated and non-aggregated species was summarized through comparative analysis of the characteristics of PrP in the absence or presence of heparin and discussed in the manuscript at Pages 17-18 Lines 338-344 and Lines 350-354. The details were as followings: The presence of 30 μg/mL heparin increased PK resistance and aggregate size of MoPrP (Figs 4 and 6), suggesting that high concentration of heparin induces a conformational change and contribute to the conversion of MoPrP to MoPrP^Sc^. As for ChPrP treated with 30 μg/mL heparin, less PK resistance and slight increase of β-sheet structure was observed. The effects of these cofactors on characteristics of ChPrP differ from those on MoPrP. These results may provide a new perspective on understanding the differences between mammalian and non-mammalian PrP and further on unraveling why prion diseases are only observed in mammals.

5 - Hep induced the formation of PrP spherical aggregates in reference 39 (supporting material), similar to what is being showed. The author should cite and compare.

Responses: Thanks for your good advices and for suggesting the important reference. We have added the comparison of PrP spherical aggregates to the revised manuscript at Page 14 Lines 265-269. The added sentences were as followings: These spherical aggregates were smaller than those reported in [38], with even more than 200 nm in size. This different was possibly due to using different heparin (30 μg/mL heparin v.s. 2 μM low molecular weight heparin) and incubating in different buffers (MilliQ water v.s. 10 mM acetate and 100 mM NaCl, pH 5.5).

Reviewer Response: ok. Review grammar of the new sentence.

Response: Thank you for your positive comments and good suggestions. We have checked all the new sentences in the revised manuscript.

6 - “Our result showed that heparin binding didn’t play a role in amyloid fibril formation under denaturation (Fig 1). However, low-molecular-weight heparin was shown to increase the stability of full-length PrPC from mice under denaturation and seeded with infected brain tissue homogenate [43]” (lines 235-238). The authors should consider differences on sample preparation: PrP:Hep interaction was in denaturing buffer (this paper) and before addition to denaturing buffer (reference 43); Hep concentration and buffer are different.

Responses: Thanks for your good advices and for suggesting the important reference. In the reference 43, LMWHep (final concentration, 25 μM) was added to the denaturing buffer containing 0.1 mg/mL rPrP (similar to this paper), or previously to the rPrP solution before rPrP was added to the denaturing buffer. The addition of LMWHep to denaturing buffer containing 0.1 mg/mL rPrP (similar to this paper) caused a delay in rPrP fibril formation when the reaction was seeded with infected mouse BH. However, our result showed that heparin addition didn’t play a role in amyloid fibril formation under denaturing conditions. This different was possibly due to using different heparin (30 μg/mL heparin v.s. 25 μM low molecular weight heparin) and incubating in different buffers (100 mM potassium phosphate buffer, 2 mol/L GuHCl, pH 6.5 v.s. 10 mM phosphate buffer, 130 mM NaCl, pH 7.4). Thus, we revised the related sentences in the revised manuscript Page 15 Lines 299-302.

Reviewer Response: ok

Response: Thank you for your positive comments.

7 - “The present study confirmed that heparin can decrease MoPrP and ChPrP stability and facilitate them to aggregate. Furthermore, our study revealed that biochemical properties of the aggregates differed depending on heparin concentrations.” (lines 247-250). The authors should be careful with this statement since inducing a decrease in solubility is not necessarily linked to a decrease in stability of the monomeric protein. Therefore, other experiments would be necessary to evaluate changes in stability.

Responses: Thanks for your good suggestions. We have revised the related sentence as suggested by reviewer. More details had been added to the revised manuscript at Page 7 Lines 124-127. The revised parts were as followings: The present study confirmed that heparin can decrease MoPrP and ChPrP solubility and facilitate them to aggregate. Furthermore, our study revealed that biochemical properties of the aggregates differed depending on heparin concentrations.

Reviewer Response: ok

Response: Thank you for your positive comments.

We hope that the reviewers and the editors are satisfactory with the above responses.

Once again, many thanks to the two reviewers and the editor.
